# Staurosporine Inhibits Frequency-Dependent Myofilament Desensitization in Intact Rabbit Cardiac Trabeculae

**DOI:** 10.1155/2012/290971

**Published:** 2012-05-09

**Authors:** Kenneth D. Varian, Brandon J. Biesiadecki, Mark T. Ziolo, Jonathan P. Davis, Paul M. L. Janssen

**Affiliations:** ^1^Department of Physiology and Cell Biology, College of Medicine, The Ohio State University, 1645 Neil Avenue, Columbus, OH 43210, USA; ^2^Department of Physiology and Cell Biology, College of Medicine, The Ohio State University, 304 Hamilton Hall, 1645 Neil Avenue, Columbus, OH 43210-1218, USA

## Abstract

Myofilament calcium sensitivity decreases with frequency in intact healthy rabbit trabeculae and associates with Troponin I and Myosin light chain-2 phosphorylation. We here tested whether serine-threonine kinase activity is primarily responsible for this frequency-dependent modulations of myofilament calcium sensitivity. Right ventricular trabeculae were isolated from New Zealand White rabbit hearts and iontophoretically loaded with bis-fura-2. Twitch force-calcium relationships and steady state force-calcium relationships were measured at frequencies of 1 and 4 Hz at 37 °C. Staurosporine (100 nM), a nonspecific serine-threonine kinase inhibitor, or vehicle (DMSO) was included in the superfusion solution before and during the contractures. Staurosporine had no frequency-dependent effect on force development, kinetics, calcium transient amplitude, or rate of calcium transient decline. The shift in the pCa_50_ of the force-calcium relationship was significant from 6.05 ± 0.04 at 1 Hz versus 5.88 ± 0.06 at 4 Hz under control conditions (vehicle, *P* < 0.001) but not in presence of staurosporine (5.89 ± 0.08 at 1 Hz versus 5.94 ± 0.07 at 4 Hz, *P* = NS). Phosphoprotein analysis (Pro-Q Diamond stain) confirmed that staurosporine significantly blunted the frequency-dependent phosphorylation at Troponin I and Myosin light chain-2. We conclude that frequency-dependent modulation of calcium sensitivity is mediated through a kinase-specific effect involving phosphorylation of myofilament proteins.

## 1. Introduction

The relationship between heart rate and myocardial contractility has been studied extensively since Bowditch first recognized what we now refer to as the force frequency relationship (FFR) [1]. Modulation of contractility through heart rate is an intrinsic property of the heart that occurs independent of neurohumoral activity and principally through augmentation of calcium handling and the altering of myofilament properties. In patients suffering from congestive heart failure (CHF), a blunted or negative FFR is observed regardless of the underlying etiology [2–4]. This alteration of normal physiology likely contributes to exercise intolerance and general lack of cardiac reserve seen in patients suffering from CHF. Although a robust increase in contractility with an increase in heart rate is a crucial regulatory property of nonfailing myocardium in all mammals [5], its governing underlying mechanisms are still incompletely understood.

Augmentation of the calcium transient amplitude and rate of decline with increased frequency has been well documented [6, 7]. The mechanism underlying altered calcium handling has been the most extensively investigated aspect of the FFR, and several mechanisms have been suggested. It is likely that the enhanced calcium handling is due in part, if not exclusively, to intrinsic properties of the calcium signaling system. An increase in heart rate increases the amount of calcium entering the L-type calcium channels per unit time and increases intracellular sodium both of which can result in an increase in sarcoplasmic reticulum (SR) load [8, 9]. The increase in SR load results in the rise in peak systolic calcium, resulting in enhanced myocardial force production. SR calcium reuptake rate increases due the sarcoplasmic reticulum calcium ATPase (SERCA2a) pump working higher on its [Ca^2+^]_i_-velocity curve. However, it is still possible (calcium-dependent) kinase(s) are activated at higher heart rates which could potentially augment calcium handling through phosphorylation of the L-type calcium channel, phospholamban, SERCA2a itself, or the ryanodine receptor. So far the most likely candidate for a frequency dependent phosphorylation is calcium calmodulin-dependent kinase II (CaMKII) which has been examined in several studies [10–12]. However, a conclusive target has yet to be found. The roles of PKC [13], PKA [14], and PKG [15] in the FFR have been investigated to some extent, but a conclusive mechanism is still lacking. Modulation of myofilament properties with changes in heart rate has been much less investigated, and the few studies that have focused on this potentially contributing mechanism have, until recently, been inconclusive. Previous studies have found myofilament calcium sensitivity to be increased [16], decreased [17], and unchanged [15] with an increase in frequency. To some extent, these differences may reside in the animal model used; *in vivo*, the rat and mouse depend much less on changes in frequency (~20% in mouse, 40–50% in rat) than larger mammals, including humans (~250–350%) [5, 18]. As such, results obtained in larger mammals may better reflect human frequency-dependent behavior. We demonstrated in rabbit right ventricular trabeculae, under near physiological conditions, that myofilament calcium sensitivity indeed decreases with frequency [19]. We then proceeded to show that, in a model of right ventricular hypertrophy [20], this frequency-dependent myofilament desensitization was impaired [21], potentially explaining diastolic dysfunction. Moreover, we showed that frequency-dependent myofilament desensitization was associated with Troponin I and Myosin light chain-2 phosphorylation [19, 21]. Lamberts and coworkers recently showed that, in failing rat myocardium, a frequency-dependent myofilament desensitization also exists [13], although they did not observe significant changes in healthy rat right ventricular trabeculae at low frequencies. Therefore, to what extent, and in particular how, myofilament calcium sensitivity changes with frequency still remains incompletely resolved.

Based on the previous studies, we hypothesize that a phosphorylation event is primarily at the basis of frequency-dependent modulation of myofilament calcium sensitivity. Using the broad-spectrum serine-threonine kinase inhibitor staurosporine, we set out to determine a potential role of kinases involved with the FFR. We show that the frequency-dependent myofilament desensitization is inhibited by staurosporine. This finding associated with an inhibition of frequency-dependent phosphorylation of myofilament proteins.

## 2. Materials and Methods

All protocols were approved by the institutional laboratory animal care and use committee of The Ohio State University. Male, New Zealand White (NZW) rabbits (1.5–2 kg) were given 5,000 U/kg heparin iv and were anesthetized with 50 mg/kg pentobarbital. The chest was opened via bilateral thoracotomy, and the heart rapidly excised and flushed retrogradely with a cardioplegic Krebs Henseleit solution containing 20 mM 2, 3 butanedione monoxime (BDM) to prevent cutting injury [22]. The right ventricle was opened and thin, uniform trabeculae were carefully dissected and mounted in a setup constructed on the stage of a Nikon-inverted fluorescent microscope as previously described [17, 19, 23, 24]. 

### 2.1. Measurement of Intracellular Calcium and Force Frequency

The cardioplegic K-H was replaced with a normal K-H solution, and the muscle was stimulated at 1 Hz, at 37°C at an extracellular calcium concentration of 1.5 mM, till contractile parameters had stabilized. The muscle was then stretched to an optimal length where further stretching raised diastolic and systolic force proportionally. It has been shown that this muscle length corresponds with a sarcomere length of around 2.2-2.3 *μ*m [25]. For loading of the indicator, temperature was temporarily dropped to 23°C to facilitate the loading process. Trabeculae were iontophoretically loaded with the calcium indicator bis-fura 2 as described previously for the indicator fura-2 [24]. The loading procedure typically lasted between 15 and 25 minutes. We have shown that the kinetics of this indicator, even at body temperature, are sufficiently fast to accurately track the calcium transient even at the highest frequency [26]. After returning to 37°C, a control force-frequency relationship was determined on each muscle by stimulating the muscle to twitch for 2 minutes (more than sufficient to reach steady state), at 1, 2, 3, and 4 Hz. Force tracings and fluorescent emissions at 340 nm and 380 nm were collected. The stimulation was returned to 1 Hz and either DMSO (0.01% v/v) or staurosporine (0.1 *μ*M) was added to the superfusion solution and allowed to circulate for 5 minutes before a second force-frequency was measured (same protocol as control). The 0.1 *μ*M concentration of staurosporine was chosen because it is at least 5 times the *in vitro K*_*i*_ for the most obvious candidate kinases (PKA 15 nM, PKC 5 nM, PKG 18 nM, CaMKII 20 nM and MLCK 21 nM) [27] while still below the concentration where some of the nonspecific effects of staurosporine have been found to occur [27].

### 2.2. Measurement of Steady-State Myofilament Activation

To obtain a steady-state myofilament calcium sensitivity relationship at 37°C, we employed potassium-induced contractures as described previously [19, 28, 29]. Immediately after the second force-frequency measurement, trabeculae under the influence of staurosporine or vehicle control were stimulated to contract at 1 or 4 Hz. The superfusion solution was switched from regular Krebs Henseleit solution to one with a modified Na/K balance (6 mM Ca^2+^ 110 mM K^+^ and 40 mM Na^+^). Bis-fura 2 fluorescent emission ratios were collected along with force till the peak of the contracture. The fluorescence signal ratio of 340/380 was converted into [Ca^2+^]_i_ by obtaining the minimum and maximum ratios (*R*_min⁡_ and *R*_max⁡_) as described previously [24]. The [Ca^2+^]_i_ values were plotted against force, and the data was fitted using the Hill equation. In each muscle, a potassium contracture was performed at 1 and 4 Hz under the influence of either staurosporine or vehicle. Muscle length was held constant between the two measurements to ensure sarcomere length was excluded as a cause in myofilament calcium sensitivity shift between the two frequencies. Therefore, paired comparisons were made between the two frequencies with or without staurosporine (*n* = 10 DMSO, *n* = 9 staurosporine).

### 2.3. Measuring Protein Phosphorylation (Pro-Q Diamond Stain)

Phosphoprotein analysis via the Pro-Q Diamond Stain was performed as described previously [19, 29]. Briefly, trabeculae twitching at either 1 or 4 Hz with either DMSO or 0.1 *μ*M staurosporine in the superfusion bath were doused with liquid nitrogen until frozen and rapidly removed from the experimental setup. The tissue was then homogenized in an SDS protein lysis buffer and loaded on a 15% 8 × 10 cm SDS-PAGE gel (1.5 mm thickness, 4% stacking gel, 15 wells). The gel was then run for 45 minutes at 175 V. The gel was fixed overnight and stained using the Pro-Q Diamond phosphoprotein stain (Invitrogen). Following staining and destaining, the gel was imaged in a Typhoon variable mode scanner (GE Healthcare) using an excitation wavelength of 532 nm and a 610 nm (BP30) emission filter at a photomultiplier setting of 450 nm. After imaging, the gel was washed in water for 1 hour and stained for total protein with Sypro Ruby protein stain (Invitrogen). Following staining and destaining, the gel was imaged in a Typhoon scanner using an excitation wavelength of 488 nm and a 610 nm (BP30) emission filter at a PMT setting of 425 nm. Densitometric analysis was performed on each band, and the ratio of Pro-Q stain intensity to total protein intensity was calculated.

### 2.4. Statistics

Data was analyzed using paired two tailed *t*-tests for sensitivity curves where *P* < 0.05 was considered significant. Average data is presented with error bars showing standard error of the mean. Two- and one-way ANOVAs with post hoc *t*-tests (Bonferroni correction) were used for analysis of twitch and calcium transient data.

## 3. Results

**Figure 1:**
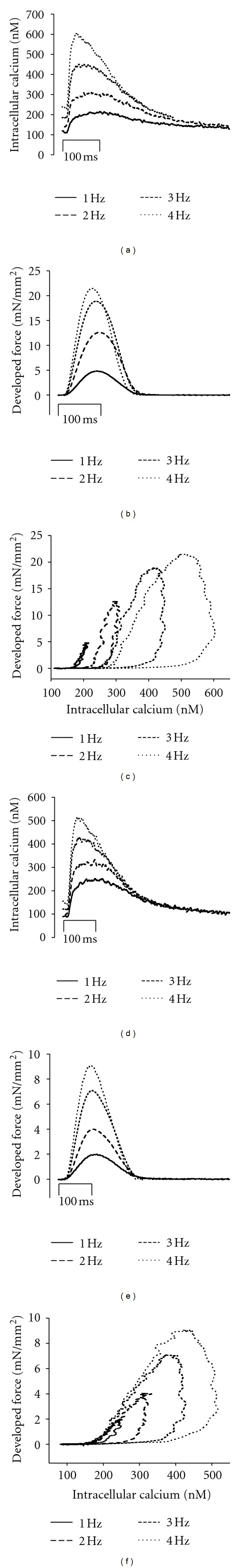
Panels (a), (b), and (c) show representative calcium transients (a), force tracings (b), and corresponding phase plane plots (c) from rabbit right ventricular trabeculae stimulated to contract at 1, 2, 3, and 4 Hz (37°C). Note the shift to the right of the relaxation trajectory of the phase plane plots. The same preparation was then treated with 100 nM staurosporine and the protocol repeated. Calcium transients (d), force tracings (e), and the resulting phase planes (f) are shown. Note the elimination of the relaxation trajectory shift after the addition of staurosporine.

Figure 1 shows an experiment where a force frequency relationship was obtained before and after the addition of staurosporine to the superfusate. As previously shown [19, 21], under control conditions, the relaxation trajectory of the phase plane plots of [Ca^2+^]_i_ versus force shifts to the right with an increase in frequency, suggesting myofilament desensitization. The phase-plane plots allow for the determination of calcium versus force relationship trajectories independent of timing [23, 30, 31]. We observed that, with the addition of staurosporine the relaxation trajectories line up, that is no shift is observed. Although this is an indirect assessment, this suggests that staurosporine was able to inhibit frequency-dependent myofilament desensitization.

**Figure 2:**
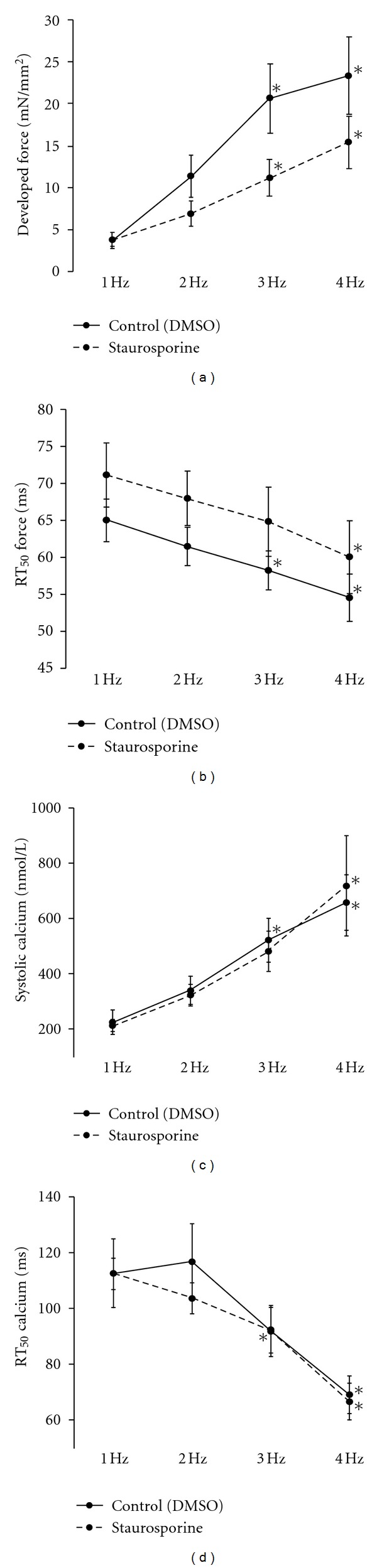
Average twitch and calcium transient data from trabeculae treated with staurosporine or vehicle (DMSO) (*n* = 8 for each group). Panels (a) and (b) show the developed force (a) and relaxation time to 90% of peak (RT_50_) at each frequency 1 through 4 Hz. Analysis by two-way ANOVA (Bonferroni correction, *P* < 0.05) showed that frequency significantly altered force and relaxation, but staurosporine did not significantly alter the FFR or FDAR. Panels (c) and (d) show the average systolic calcium levels (c) and time constant of calcium decline (tau). There was no significant effect of 100 nM staurosporine, nor an interaction between staurosporine and frequency. The effect of frequency within each group was analyzed by one-way AVOVA (*significantly different from 1 Hz).

Figure 2 shows average data for developed force (*F*_dev_), time from peak tension to 50% relaxation (RT_50_ force), systolic calcium concentration (nM), and the RT50 of calcium decline (RT_50_ calcium). Developed force increased with frequency in both control and staurosporine groups when stimulation frequency is increased. Statistical analysis (2-way ANOVA) revealed no significant effect of staurosporine on any force, or calcium-transient parameter.

**Figure 3:**
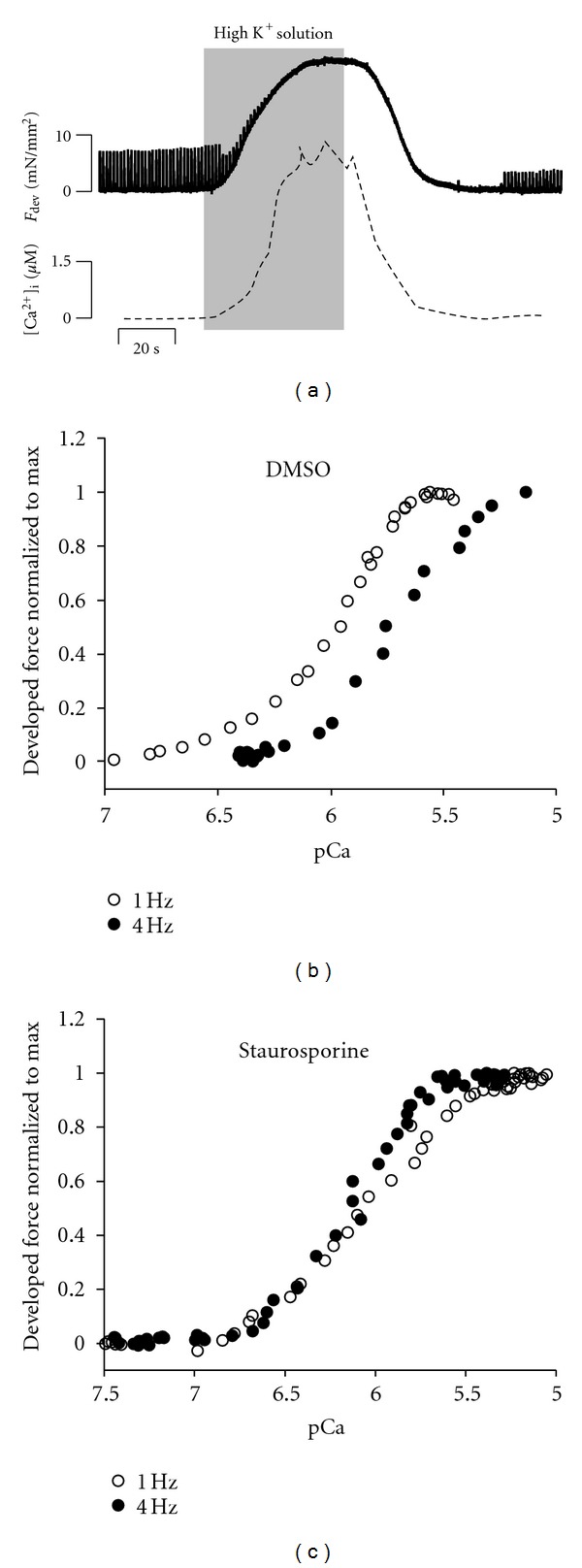
Figure 3 shows a representative acquisition and analysis of the force-calcium relationship. Panel (a) shows the chart of a potassium contracture. The trabecula in this example is twitching at 1 Hz (top of panel) when the superfusate solution is rapidly switched from normal Krebs Henseleit solution to that with a higher potassium and lower sodium concentration. Diastolic calcium (lower panel) is measured by rapidly switching the excitation wavelength between 340 and 380 nm. After the potassium solution is washed out, the contracture relaxes and the muscle recovers. Panels (b) and (c) show representative force pCa relationships obtained in trabeculae twitching at 1 or 4 Hz with vehicle (b) or staurosporine (c) in the superfusate solution.

Next, we set out to determine if staurosporine had an effect on myofilament calcium sensitivity modulations due to frequency. For this, we employed so called “potassium contractures” to introduce a pseudo-steady state between the intracellular calcium concentration and developed force in order to measure the calcium sensitivity of the myofilaments.  Figure 3(a) shows the original data of a representative potassium contracture. The superfusate solution is rapidly switched from normal Krebs-Henseleit solution to one containing high potassium and low sodium (indicated by the grey area in the figure). Upon application of the “potassium contraction” solution, calcium enters the cytoplasm slowly, inducing a slowing forming contracture. By rapidly switching between 340 nm and 380 nm excitation wavelengths, diastolic calcium was measured as this contracture formed, up till peak force had developed. Thereafter, the perfusate was switched back to the normal Krebs-Henseleit solution, and the membrane potential slowly reestablished, while calcium is removed from the cytosol, and subsequently the contracture dissipates. The simultaneously recorded force and bis-fura-2 ratio data allows us to construct a myofilament calcium sensitivity relationship, from which characterizing parameters are determined by fitting this data with the Hill equation. Figures 3(b) and 3(c) show an example of [Ca^2+^]_i_ versus. force curves derived from control and staurosporine protocols. A potassium contracture was performed at 1 and 4 Hz for each muscle. 

**Figure 4:**
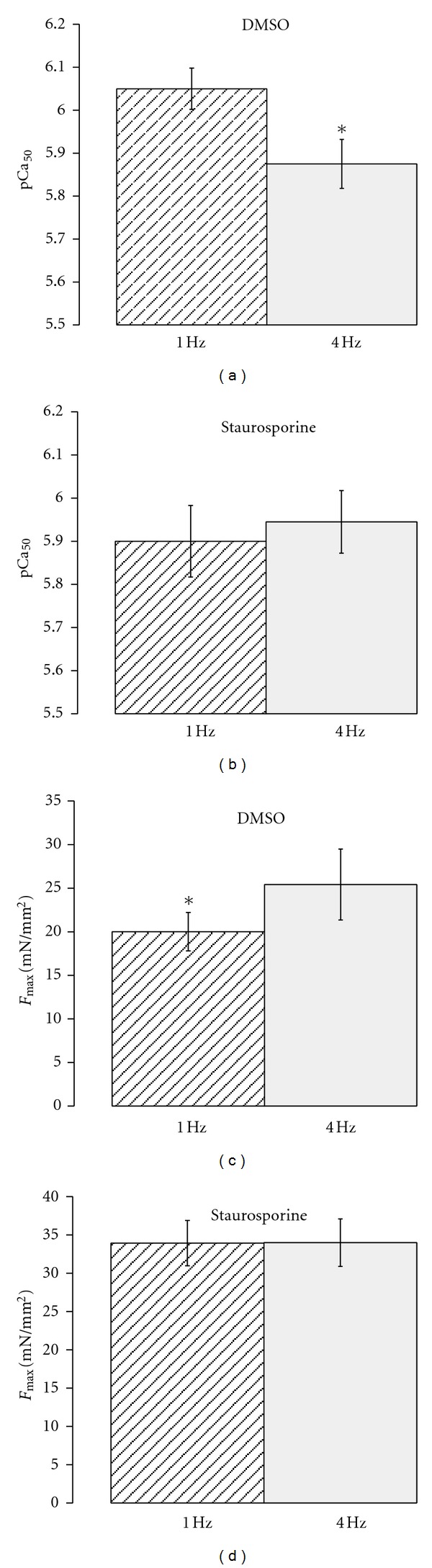
Average pCa_50_ and maximum force (*F*_max⁡_) data obtained from the force calcium relationships (*n* = 10 for DMS and *n* = 9 for staurosporine). A significant difference (*P* < 0.05 denoted with *) was seen in the pCa_50_ and *F*_max⁡_ between the 1 and 4 Hz under control conditions (panels (a) and (b)). These shifts in sensitivity and *F*_max⁡_ were eliminated by staurosporine (panels (c) and (d)).

Figure 4 shows the average for parameters pCa_50_ and *F*_max⁡_ for control and staurosporine. While there was a significant desensitization with respect to the pCa_50_ from 1 to 4 Hz in the control group, in line with our previous observations, no such frequency-dependent shift was detected with staurosporine. Maximum developed force of each potassium contracture (*F*_max⁡_) was not significantly (*P* > 0.05) different between each group although the difference in average *F*_max⁡_ between 1 and 4 Hz in the control group approached significance (*P* < 0.1). The hill coefficient, an indicator of cooperativity of the myofilament force response, was not significantly different between control and staurosporine sensitivity curved (*N*_Hill_; control: 1 Hz 3.99 ± 0.60, 4 Hz 2.82 ± 0.56; staurosporine: 1 Hz 2.28 ± 0.38, 4 Hz 2.0 ± 0.18).

**Figure 5:**
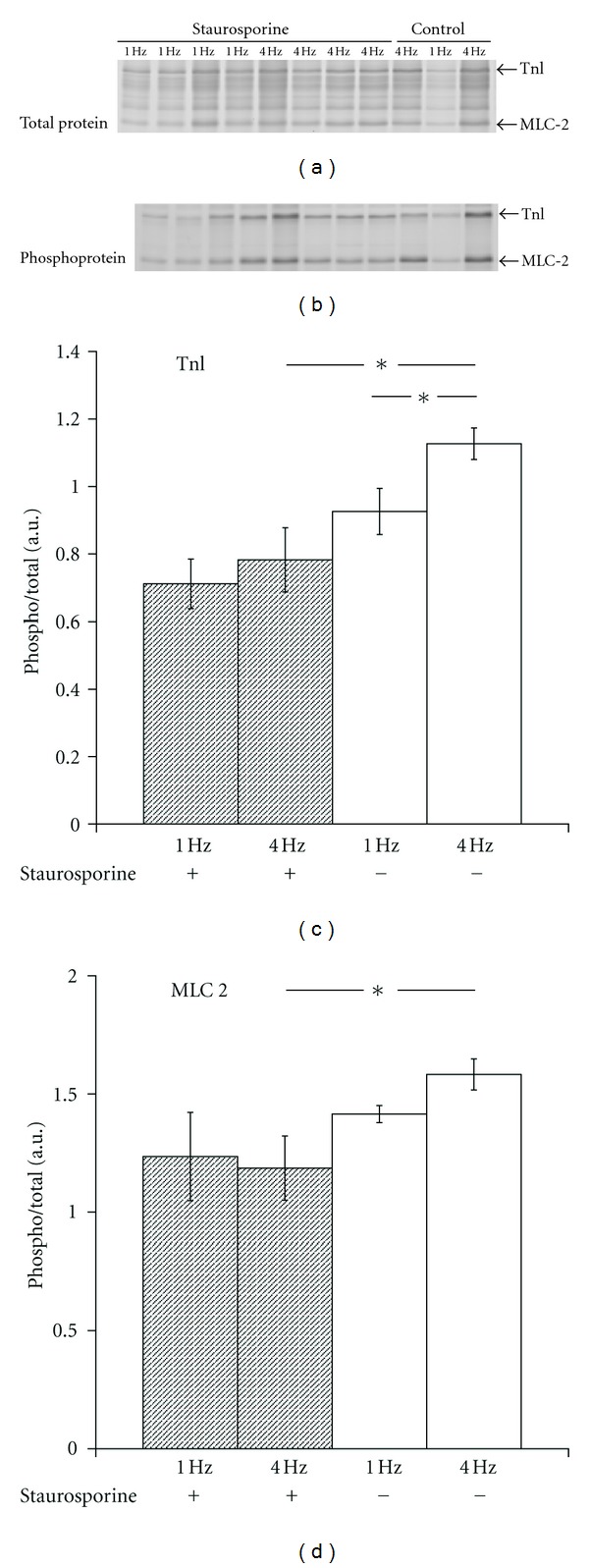
Analysis of TnI and MLC-2 phosphorylation status in muscles stimulated to twitch at 1 or 4 Hz, with and without staurosporine. Panel (a) shows a representative SDS gel stained for total protein using the Sypro Ruby protein stain. Panel (b) shows the same gel counterstained for phosphoprotein using the Pro-Q Diamond Stain. Densitometric analysis of bands corresponding to TnI and MLC-2 was obtained from each stain and the ratio of phosphoprotein to total protein calculated for each group. TnI phospho/total protein ratio is shown in panel (c) and MLC-2 shown in panel (d) (*n* = 4 for each group, other gel not shown); error bars are SEM, *P < 0.05 considered significant.

Figure 5 shows a section of an SDS gel stained for total protein (a) (Sypro ruby) and phosphoprotein (b) (Pro-Q Diamond) at the location corresponding with Troponin I (TnI) and Myosin light chain-2 (MLC-2). The average ratio of band density of phosphoprotein to total protein for TnI (c) and MLC-2 (d) is also depicted. A significant increase in the ratio was found for TnI between 1 and 4 Hz in the control group consistent with previous findings [19]. The difference in ratio between 4 Hz with staurosporine and 4 Hz with DMSO was also found to be significantly different. The difference in ratio of phosphoprotein to total protein for MLC-2 approached significance between 1 and 4 Hz for the control group (*P* < 0.1 but >0.05). However, a significant difference in MLC-2 phosphorylation was found between 4 Hz staurosporine and 4 Hz DMSO.

## 4. Discussion

In this study, we investigated whether frequency-dependent myofilament desensitization is mediated by a kinase dependent pathway and how this phenomenon impacts cardiac trabecular twitch contractions. We now show for the first time that broad-spectrum serine-threonine kinase inhibition inhibits frequency-dependent myofilament desensitization. Staurosporine was able to inhibit frequency-induced phosphorylation of TnI which is likely (at least partially) responsible for frequency-dependent myofilament desensitization. We also show that staurosporine does not, at the concentration used in our studies, affect the dynamics of the calcium transient or its relationship to stimulation rate. From this, we conclude serine threonine kinase pathways activated with changes in frequency mainly, if not exclusively, modulate myofilament function, but not calcium transient regulation. Although changes in phosphatase activity cannot be excluded at this point, the near-complete abolishment of frequency-dependent changes in myofilament calcium sensitivity indicate that phosphatases likely play only a minor, if any, role.

Despite extensive research, the molecular mechanisms of frequency-dependent augmentation calcium handling and modulations of myofilament calcium sensitivity have eluded full understanding. This is not entirely surprising, as frequency modulation occurs *in vivo* during a highly dynamic response. Governing factors such as calcium concentration, external mechanical load, internal passive-elastic elements, and ionic fluxes never reach a steady state balance. Thus, a steady-state snap shot of these parameters or interactions can never fully describe the prevailing dynamical situation. Using our approach, we found that staurosporine, at a concentration where several major kinases are inhibited, had no effect on the frequency dependency of the calcium transient amplitude or decline, nor force amplitude or decline. This data suggests that changes in frequency do not induce phosphorylations that affect the calcium transient itself. This finding is consistent with Kassiri et al. where the use of broad spectrum kinase inhibitor K252-a yielded no change in the frequency-dependent augmentation of calcium handling [15]. Although we cannot exclude phosphorylation events do occur with frequency on key calcium handling proteins, our results indicate that, if they do occur, their functional effect on the cytosolic calcium transient appears to have little or no functional significance. Other studies have focused on the specific effect of CaMKII [11, 32] as a potential regulator of calcium handling dynamics. Most recently, Picht and coworkers [12] found that CaMKII inhibition directed to the SR inhibits frequency-dependent acceleration of calcium decline by inhibiting, an increase in SERCA2a *V*_max⁡_. While, in the present study we did not measure or derive SERCA velocity measurements, we did find that the RT_50_ of calcium decline was not different between control and staurosporine. This thus suggests that intracellular calcium decline, which can be impacted by the calcium transient amplitude itself [33], was not significantly blunted with kinase inhibition under the present experimental conditions, which were very close as they prevail *in vivo* regarding mechanical load, temperature, and stimulation range. Clearly, our data here suggest that the intrinsic properties, rather than frequency-induced phosphorylations, of the calcium handling system play a major (if not the only) role in augmentation of amplitude and rate of decline.

A striking effect of staurosporine was the complete elimination of the frequency-dependent shift in myofilament calcium sensitivity from 1 to 4 Hz. In addition, there appears to (possibly) be an effect of frequency on maximal developed force. Although this shift in *F*_max⁡_ did not quite reach significance (*P* < 0.1 but >0.05), there may still be a kinase-dependent effect on *F*_max⁡_. Supporting this speculation is the fact that in the staurosporine group, there was almost no variability in the myofilament sensitivity curve *F*_max⁡_. These data show that changes in stimulation frequency activate signaling pathways that lead to the activation of protein kinases, which in turn affect myofilament calcium sensitivity and possibly *F*_max⁡_. Since these effects were completely eliminated by staurosporine, it is likely that phosphorylation events comprise most, if not all, of the mechanism responsible for frequency-dependent changes in myofilament function. This data was verified using a phosphoproteomics protocol. We show that TnI is hyperphosphorylated at higher frequency, in line with our previous observation [19]. Staurosporine inhibited this hyperphosphorylation, and we observed that the relative TnI phosphorylation levels were lower than those in the absence of staurosporine at 1 Hz, indicating that even, at the lowest frequency baseline, phosphorylation of TnI is present. Despite the clear effect of staurosporine on myofilament calcium sensitivity, staurosporine induced no significant interaction between frequency and the twitch parameters measured. Frequency-dependent acceleration of relaxation (FDAR) was still mostly intact. This paradigm may be explained by the fact that staurosporine may have inhibited more than one pathway which can have opposite effects on myofilament calcium sensitivity and twitch parameters. We found that staurosporine significantly reduced TnI and MLC-2 phosphorylation at 4 Hz, compared to control but not at 1 Hz. It has been shown by many investigators that TnI phosphorylation can decrease myofilament calcium sensitivity [34–36]. Also, it has been shown that MLC-2 phosphorylation can increase myofilament calcium sensitivity [37–39]. Therefore, we should not necessarily expect the effect of staurosporine on the twitch parameters to be straightforward. The pCa_50_ values attained by potassium contractures reveal that staurosporine, while inhibiting the shift in pCa_50_, set the myofilament sensitivity to level between that of 1 and 4 Hz in the control group 4 Hz. With sensitivity set to a slightly lower value, the rate-limiting step of relaxation may begin to favor other factors such as the rate of calcium decline, including Troponin-C calcium off-rate kinetics [40, 41] and calcium-regulated SERCA-activity [33]. In addition, MLC-2 phosphorylation has been shown to slow cross bridge kinetics [42] which would counter the acceleratory effect of TnI phosphorylation. Abolishment of both of these may have a null effect on relaxation. Finally, it has been previously speculated shifts in myofilament calcium sensitivity do not influence the actual rate of relaxation to a large extent [43], and recent views on contraction-relaxation coupling have postulated an overall governing mechanical rate that may impact contractile kinetics as a whole and not be specific to relaxation [44, 45].

Although for certain compounds specific inhibitors exist that work well in a test tube with a limited number of substrates, recent pilot experiments revealed that compounds such as for instance ML7 (an inhibitor of MLC2) have drastic side effects on the calcium transients, and thus likely have multiple targets in the myocyte, to the extent that the muscle barely contracts, and prohibited an unambiguous investigation of contractile properties in present of this compound. As the study of frequency-dependent activation requires an intact preparation, future studies in which the specificity of kinases is extensively documented in such an integrated preparation may aid to reveal the exact identification of the specific kinase responsible. For instance, CaMKII could be a potential mediator, since the increase in calcium during high frequencies may activate this pathway. On the other hand, the time course of CaMKII activation may be too slow [46] to be critically involved in frequency-dependent desensitization, but, at present, the complete elucidation of pathway and specific amino acid targets is deemed beyond the scope of this study. Furthermore, we and others showed the importance of frequency-dependent as well as the role of TnI in hypertrophy [21] and aging [47], and thus future elucidation of specific kinases and specific targets would be of significant importance, as would be to resolve the temporal resolution of the changes in myofilament responsiveness upon a change in frequency.

In summary, we have shown that broad-spectrum kinase inhibition has no significant effect on calcium transient amplitude and rate of decline, or how these parameters change with frequency. Frequency-dependent shifts in myofilament calcium sensitivity are however virtually abolished with staurosporine, suggesting the kinase-dependent augmentation of the force frequency appears to primarily target the myofilaments, whereas the frequency-dependent augmentation of the calcium transient mainly relies on its intrinsic properties.
